# The Nephroprotective Efficacy of Omega-3 Fatty Acids Against Streptozotocin-Induced Diabetic Renal Injury: A Biochemical, Histopathological and Ultrastructural Study

**DOI:** 10.3390/antiox15080975

**Published:** 2026-08-06

**Authors:** Emrah Zayman, Eda Nur Özsoy, Mehmet Erman Erdemli, Zeynep Erdemli, Nilüfer Bulut, Feyza İnceoğlu, Mehmet Gül

**Affiliations:** 1 Department of Histology and Embryology, Faculty of Medicine, Malatya Turgut Özal University, Malatya 44210, Türkiye; 2 Department of Histology and Embryology, Faculty of Medicine, Inonu University, Malatya 44280, Türkiye; 3 Department of Medical Biochemistry, Faculty of Medicine, Inonu University, Malatya 44280, Türkiye; 4 Department of Nutrition and Dietetics, Faculty of Health Sciences, Malatya Turgut Özal University, Malatya 44210, Türkiye; 5 Department of Biostatistics, Faculty of Medicine, Malatya Turgut Özal University, Malatya 44210, Türkiye

**Keywords:** diabetic nephropathy, omega-3 fatty acids, caspase-3, podocytes, oxidative stress, ultrastructure

## Abstract

The present study was designed to evaluate the structural and biochemical efficacy of Omega-3 fatty acids in preventing renal injury in streptozotocin (STZ)-induced diabetic kidney injury and to correlate systemic oxidative stress parameters with histological and ultrastructural parameters. Twenty-eight male Wistar Albino rats were randomly divided into four groups (*n* = 7) as follows: Control, Omega-3 (500 mg/kg, orally), Diabetes Mellitus (DM; 50 mg/kg STZ, i.p.), and DM+Omega-3 (STZ, followed by 500 mg/kg Omega-3 for 42 days). Biochemical analysis of renal function (blood urea nitrogen (BUN) and creatinine) and oxidative status (malondialdehyde (MDA), glutathione (GSH), superoxide dismutase (SOD), catalase (CAT), total antioxidant status (TAS), total oxidant status (TOS) and oxidative stress index (OSI)) was measured by ELISA. Histopathological scoring and Caspase-3 staining were performed by Hematoxylin and Eosin (H&E) and immunohistochemically, respectively. Transmission Electron Microscopy (TEM) was used to examine the renal tissues. Our results showed that the DM group presented with severe hyperglycemia, uremia, and a significant oxidative shift, as evidenced by increased MDA/TOS levels and depleted antioxidant defenses. Light microscopy showed extensive glomerular damage, tubular degeneration and inflammatory infiltration in the kidneys of diabetic rats. Immunohistochemical analysis revealed strong Caspase-3 expression in the tubular and glomerular compartments. TEM showed severe podocyte effacement and damage to the glomerular basement membrane (GBM). Notably, Omega-3 supplementation significantly reversed renal dysfunction, restored the pro-oxidant/antioxidant balance and reduced histopathological injury scores. Moreover, Omega-3 treatment efficiently blocked Caspase-3-mediated apoptotic signaling and preserved the ultrastructural integrity of the glomerular filtration barrier (GFB) and tubular mitochondria. Our results demonstrate the potent nephroprotective effects of Omega-3 fatty acids in reducing oxidative stress, preventing programmed cell death and maintaining the stability of the renal parenchyma’s microarchitecture. These findings suggest that Omega-3 fatty acids could be an effective adjuvant therapeutic agent for the management of diabetic nephropathy.

## 1. Introduction

Diabetes mellitus (DM) is a global metabolic epidemic. The most common microvascular complication is diabetic nephropathy (DN), which is the leading cause of end-stage renal disease (ESRD) worldwide [1]. DN is characterized by thickening of the glomerular basement membrane, mesangial expansion and progressive tubular atrophy. This leads to an irreversible reduction in the glomerular filtration rate (GFR), as well as elevated systemic levels of blood urea nitrogen (BUN) and creatinine [2]. While the etiology of diabetic renal injury is multifactorial, most current histological and biochemical evidence suggests that metabolic derangements induced by persistent hyperglycemia are the main disruptors of the renal filtration barrier and tubular epithelial architecture [3].

A fundamental mechanism driving the pathogenesis of tubular and glomerular degradation in diabetes is the significant increase in oxidative stress [4]. In the diabetic milieu, the excessive production of reactive oxygen species (ROS) overloads the body’s natural antioxidant defenses, resulting in a critical redox imbalance [5]. This state is characterized by the depletion of essential antioxidant enzymes, such as superoxide dismutase (SOD) and catalase (CAT), as well as reduced levels of reduced glutathione (GSH) [6]. The subsequent oxidative burden promotes extensive lipid peroxidation, as evidenced by elevated malondialdehyde (MDA) levels, and significantly increases the total oxidant status (TOS) and the oxidative stress index (OSI) in the renal tissue [7]. These pro-oxidant events act as upstream triggers of downstream inflammatory cascades and programmed cell death [8].

A final common pathway for the loss of renal cellularity in DM is apoptosis, which is primarily mediated by the cysteinyl aspartate-specific protease (Caspase) family [9]. Specifically, the activation and overexpression of Caspase-3 in the renal tubular epithelium and glomeruli are the major histological hallmarks of irreversible cellular injury and structural collapse in DN [9]. Thus, therapeutic approaches that reduce oxidative stress and inhibit the Caspase-3 apoptotic pathway are essential for preserving the histological and ultrastructural integrity of diabetic kidneys [10].

Omega-3 polyunsaturated fatty acids (PUFAs), particularly eicosapentaenoic acid (EPA) and docosahexaenoic acid (DHA), exhibit strong pleiotropic properties and possess significant antioxidant, anti-inflammatory and cytoprotective activities [11]. Omega-3 PUFAs are incorporated into lipid bilayers, influencing membrane fluidity and decreasing oxidative stress-induced structural damage. They also reduce the activity of pro-apoptotic pathways in various metabolic disturbance models [12]. However, the extent to which Omega-3 supplementation can restore the specific histological integrity, particularly in the proximal and distal tubules, and inhibit apoptotic signaling in an STZ-induced diabetic model must be determined through detailed analysis.

This study aimed to evaluate the structural and biochemical nephroprotective efficacy of Omega-3 fatty acids in streptozotocin (STZ)-induced diabetic injury. By correlating systemic and tissue-specific oxidative stress parameters (MDA, GSH, SOD, CAT, total antioxidant status (TAS), TOS and OSI) with rigorous histopathological scoring and Caspase-3 immunohistochemistry, our study provides a comprehensive multimodal understanding of the capacity of Omega-3 to attenuate DN.

## 2. Materials and Methods

### 2.1. Animals

Twenty-eight 12-week-old male Wistar Albino rats weighing 200–300 g were randomly divided into four experimental groups and housed at the İnonu University Experimental Animal Research Center. The animals were maintained under standard laboratory conditions (21 ± 1 °C, 50–55% relative humidity and a 12 h light/dark cycle) and had free access to standard rodent chow and water. All experimental procedures were performed in accordance with the ethical principles of animal research and were reviewed and approved by the Local Ethics Committee for Animal Experiments of Inonu University (Protocol No. 2022/12-2; Haybis No. 15429).

### 2.2. Experimental Design and Animal Groups

DM was induced in rats by a single intraperitoneal injection of STZ (Cayman Chemicals, Ann Arbor, MI, USA) dissolved in physiological saline (0.9% NaCl) at a dose of 50 mg/kg body weight [13]. Diabetes were considered present if blood glucose levels were at least twice as high on the 4th day after injection, after which the 42-day experimental period began. Body weight and blood glucose levels were regularly measured at 14-day intervals in the rats. Omega-3 treatment was performed using EFA Liquid (280 mg/mL Omega-3; 85 mg/mL EPA; 148 mg/mL DHA).

Twenty-eight male rats were randomly divided into 4 groups of seven:

- Control group: Received standard chow and water ad libitum.
- Omega-3 group: Received 500 mg/kg of Omega-3 fatty acids orally for 42 days.
- DM group: Rats were injected intraperitoneally with STZ, 50 mg/kg in saline once to induce diabetes.
- DM+Omega-3 group: Rats were treated with 500 mg/kg of Omega-3 orally for 42 days after diabetes was induced with STZ (50 mg/kg, intraperitoneally).

### 2.3. Biochemical Analysis of Renal Tissue

At the end of the experimental period, the rats were anesthetized with an intraperitoneal injection of 5 mg/kg xylazine and 50 mg/kg ketamine. While under deep anesthesia, blood samples were collected from the posterior vena cava via a midline abdominal incision, and euthanasia was completed by exsanguination. The kidneys were removed and the tissue was immediately homogenized in a phosphate-buffered solution (1:9 *w*/*v*) using a tissue homogenizer (IKA-Werke GmbH & Co. KG, Staufen in Breisgau, Germany) at 14,000 rpm for 1 min at 4 °C. MDA levels were directly assayed in the homogenates. The homogenates were centrifuged at 600× *g* for 20 min at 4 °C to obtain the supernatant for further enzymatic assays. Levels of GSH, SOD, CAT, TAS and TOS were then determined as described previously [14]. The oxidative stress index (OSI) was calculated by dividing the TOS level by the TAS level.

For MDA analysis, the tissue homogenates were reacted with 1% phosphoric acid and 0.6% thiobarbituric acid at 100 °C for 40 min. After cooling, n-butanol was added to extract the chromogen, after which the absorbance was measured at 535 nm. The supernatants were deproteinized by tricarboxylic acid (TCA) and then centrifuged at 4500 rpm for 25 min to remove the precipitate and determine the level of reduced GSH. The protein-free supernatant was then treated with DTNB and Na_2_HPO_4_, after which the absorbance was measured at 410 nm. The MDA and GSH levels were expressed as nmol/g of wet tissue weight.

Serum was separated from blood samples by centrifugation at 600× *g* for 15 min at 4 °C. Serum BUN and creatinine concentrations were measured using commercially available assay kits (Abbott, Abbott Park, IL, USA) and the enzymatic colorimetric method on an automated biochemical analyzer (Architect C8000, Abbott Laboratories, Abbott Park, IL, USA).

### 2.4. Histological and Immunohistochemical Staining

The removed renal tissues were placed immediately in 10% neutral buffered formalin (NBF) and fixed for 24 h at a controlled room temperature of (22–24 °C). The specimens were fixed and processed appropriately through a routine dehydration cascade in a graded series of ethanol, cleared in xylene, and embedded in paraffin wax blocks. To evaluate general morphological integrity, 5–6 μm thick serial sections were prepared using a rotary microtome and stained with H&E using standard laboratory protocols.

To evaluate the extent of apoptotic activity, 4 μm-thick paraffin sections were mounted on coated slides and subjected to immunohistochemical labeling. Antigen retrieval was performed in a specialized pressure cooker system (Retriever 2100, Prestige Medical Ltd., Blackburn, UK) using a citrate buffer at 120 °C for 15 min. After cooling, endogenous peroxidase activity was blocked with 3% hydrogen peroxide for 10 min. The sections were then incubated for 5 min in Protein V blocking solution to prevent non-specific binding. The slides were then incubated with primary monoclonal anti-Caspase-3 antibody (p17 Antibody (D-12), sc-373730; Santa Cruz Biotechnology, Inc., Dallas, TX, USA) diluted 1:300 for 1 h at room temperature. Signal amplification was performed using a horseradish peroxidase detection kit (Santa Cruz Biotechnology, Inc., Dallas, TX, USA) involving a 10 min incubation with a biotinylated goat anti-polyvalent secondary antibody, followed by a further 10 min incubation with streptavidin-peroxidase. Immunoreactivity was visualized following 15 min of exposure to the AEC chromogen substrate. Intermediate washing steps with PBS were performed throughout the protocol, except for the protein blocking step and the primary antibody application.

An experienced histologist, who was unaware of the experimental groups, performed the microscopic evaluations. Images were captured and a quantitative histological analysis was performed using an Eclipse Ni-U light microscope equipped with a DS-Fi3 (Nikon Instruments Inc., Melville, NY, USA) digital camera and NIS-Elements 5.02 documentation and image analysis software (Nikon Corporation, Tokyo, Japan).

Renal parenchymal injury was systematically scored on the basis of parameters of glomerular, tubular and inflammatory injury. Glomerular damage, characterized by structural dilatation, obliteration of Bowman’s space and cellular lysis or necrosis, was semi-quantitatively assessed using a 4-point scale as follows: 0 (normal morphology), 1 (mild lysis with dilatation of the glomerular basement membrane and closure of Bowman’s space), 2 (moderate lysis); and 3 (diffuse lysis or necrosis). One hundred glomeruli were scored and evaluated in each kidney section. The maximum total glomerular damage score was 300. In addition, pathological changes in the proximal tubules and distal tubules were graded according to the percentage of affected tubules: 0 (no injury), 1 (<25% affected), 2 (25–50% affected) and 3 (>50% affected). The interstitial infiltrate of inflammatory cells was scored as follows: 0 (absent), 1 (mild), 2 (moderate), or 3 (severe). The maximum total damage score was 9.

The immunohistochemical expression of Caspase-3 as a biomarker of apoptotic activity in the renal parenchyma was assessed. The histological score (H-score) method was employed to quantify the level of Caspase-3-positive immunoreactivity in the glomerular and tubular (proximal and distal) compartments. The semi-quantitative scoring system was calculated by multiplying the percentage cells stained positively by the weighted staining intensity, using the following formula: H-score = ΣPi (i + 1), where Pi is the percentage (0–100%) of cells stained in each intensity category. Immunostaining intensity was scored as follows: 0, no detectable immunoreactivity; 1, weak immunoreactivity detectable above background/control levels; 2, distinct/moderate immunoreactivity; and 3, strong immunoreactivity. H scores ranged between 0 and 400.

### 2.5. Statistical Analysis

Data were statistically analyzed using GraphPad Prism 9 for Mac OS X (GraphPad Software, San Diego, CA, USA). Descriptive statistics are presented as mean ± SD and median. The normality of distribution was assessed by the Shapiro–Wilk test. The statistical significance of differences between the four groups was calculated using one-way ANOVA with Tukey’s multiple comparison test. For all statistical analysis, differences were considered to be significant when *p* < 0.05.

## 3. Results

### 3.1. Glycemia Temporal Profiles and Systemic Metabolic Assessment

**Figure 1:**
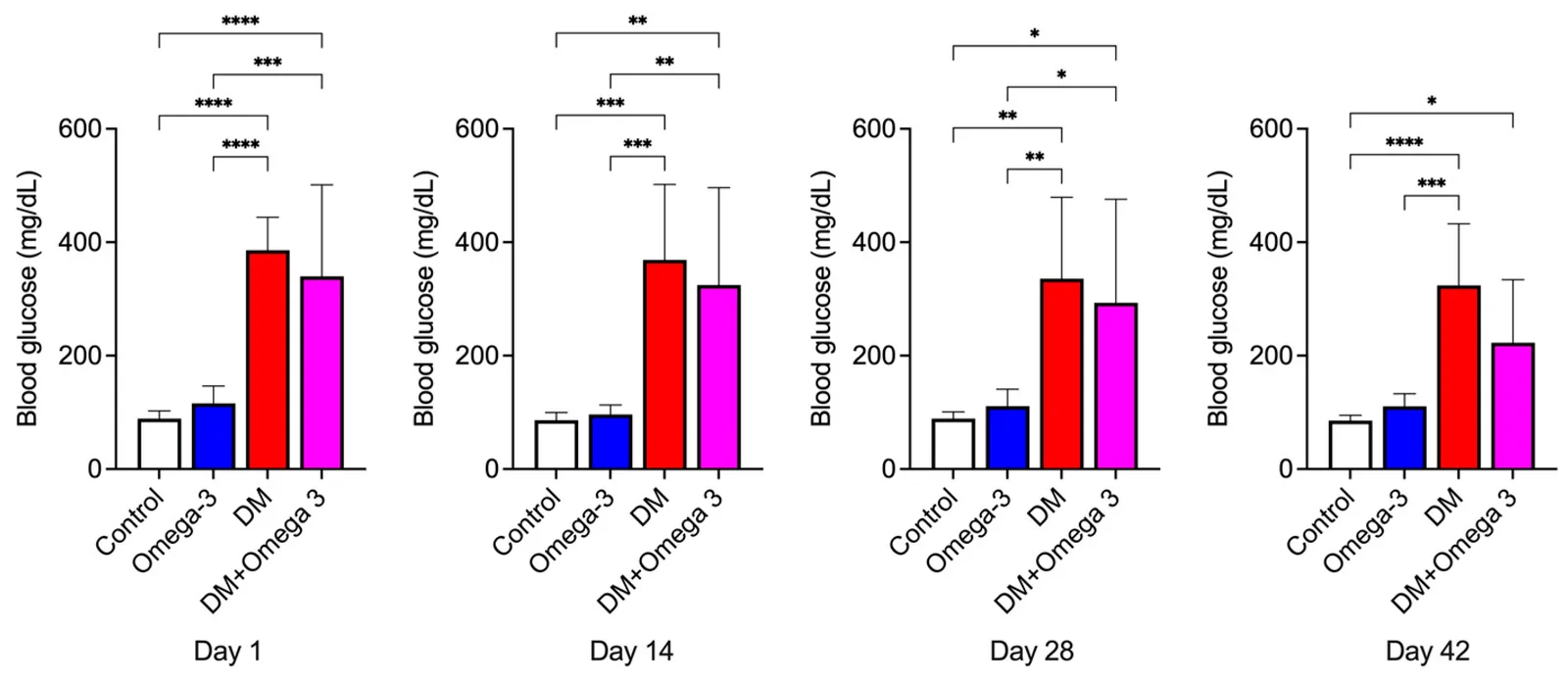
Mean blood glucose levels at each time point for the different groups. Error bars represent the SD. Statistical analysis was performed using one-way ANOVA followed by Tukey’s post-test, with *p* values comparing each pair of experimental groups shown in the graphs. * *p* < 0.05, ** *p* < 0.01, *** *p* < 0.001, **** *p* < 0.0001.

Diabetes was confirmed in the experimental animals in our previous study following STZ administration and our findings demonstrated the successful induction of diabetes [15] (Figure 1). A longitudinal assessment of blood glucose levels over 42 days revealed distinct metabolic patterns among the groups (Figure 1). At baseline (Day 1), both the DM and DM+Omega-3 groups exhibited severe hyperglycemia (median 374 mg/dL and 352 mg/dL, respectively), which was significantly higher than in the euglycemic Control (85 mg/dL) and Omega-3 (113 mg/dL) groups. The untreated DM group exhibited severe and persistent hyperglycemia throughout the experiment, with no significant temporal variation. In contrast, the DM+Omega-3 group had statistically higher blood glucose levels than healthy controls at all time points. There was also a significant reduction in blood glucose levels from Day 14 (375 mg/dL) to Day 42 (180 mg/dL) in the group, but this difference was not statistically significant due to high intra-group variance. Meanwhile, the Control group remained metabolically stable and the Omega-3 group was physiologically normoglycemic, although with minor temporal fluctuations. Intergroup comparisons revealed highly significant differences at all studied time points.

### 3.2. Biochemical Parameters and Oxidative Stress Status

**Figure 2:**
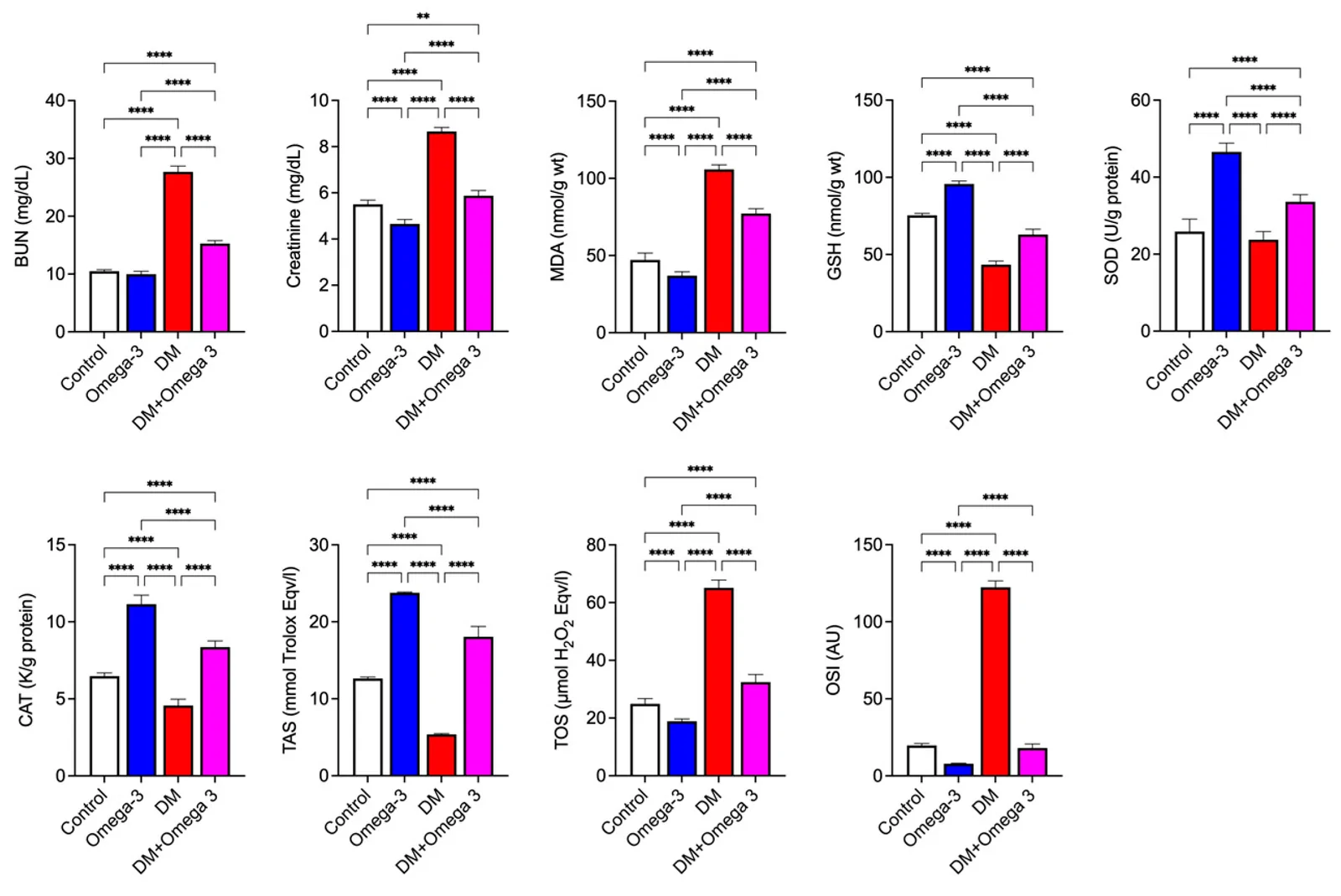
Comparison of oxidant/antioxidant and oxidative stress parameters, as well as serum parameters among the groups. Error bars represent the SD. Statistical analysis was performed using one-way ANOVA followed by Tukey’s post-test, with *p* values comparing each pair of experimental groups shown in the graphs. ** *p* < 0.01, **** *p* < 0.0001.

Serum parameters and tissue oxidative markers were analyzed to evaluate the degree of systemic renal dysfunction and local oxidative stress load induced by hyperglycemia. Biochemical analysis showed a significant reduction in the renal filtration capacity caused by DM. Compared with the Control group, significant increases in BUN (27.67 ± 1.01 mg/dL) and creatinine (8.66 ± 0.17 mg/dL) levels were demonstrated (*p* < 0.05). However, Omega-3 supplementation (the DM+Omega-3 group) was found to efficiently attenuate this uremic profile, significantly reducing the BUN and creatinine levels to within the physiological range (15.27 ± 0.52 and 5.87 ± 0.23 mg/dL, respectively), which suggests the preservation of the glomerular filtration rate (Figure 2).

The DM group showed a marked lipid peroxidation in terms of the pro-oxidant/antioxidant balance, with significant increases in MDA (105.76 ± 2.98 nmol/qwt) and TOS (65.13 ± 2.65 µmol H_2_O_2_ Eqv/L). An oxidative shift was also detected by a significant increase in OSI, reaching 122.36 ± 4.19 in diabetic rats. The opposite was observed in the DM group, where the endogenous antioxidant system was significantly compromised, resulting in the depletion of GSH levels and a decrease in SOD and CAT activities. Interestingly, Omega-3 administration to the DM+Omega-3 group exerted a potent antioxidant effect, significantly suppressing the MDA and TOS levels and restoring the enzymatic (SOD and CAT) and non-enzymatic (GSH) antioxidant defenses (*p* < 0.05) (Figure 2).

### 3.3. Histopathological Evaluation of Renal Tissue

**Figure 3:**
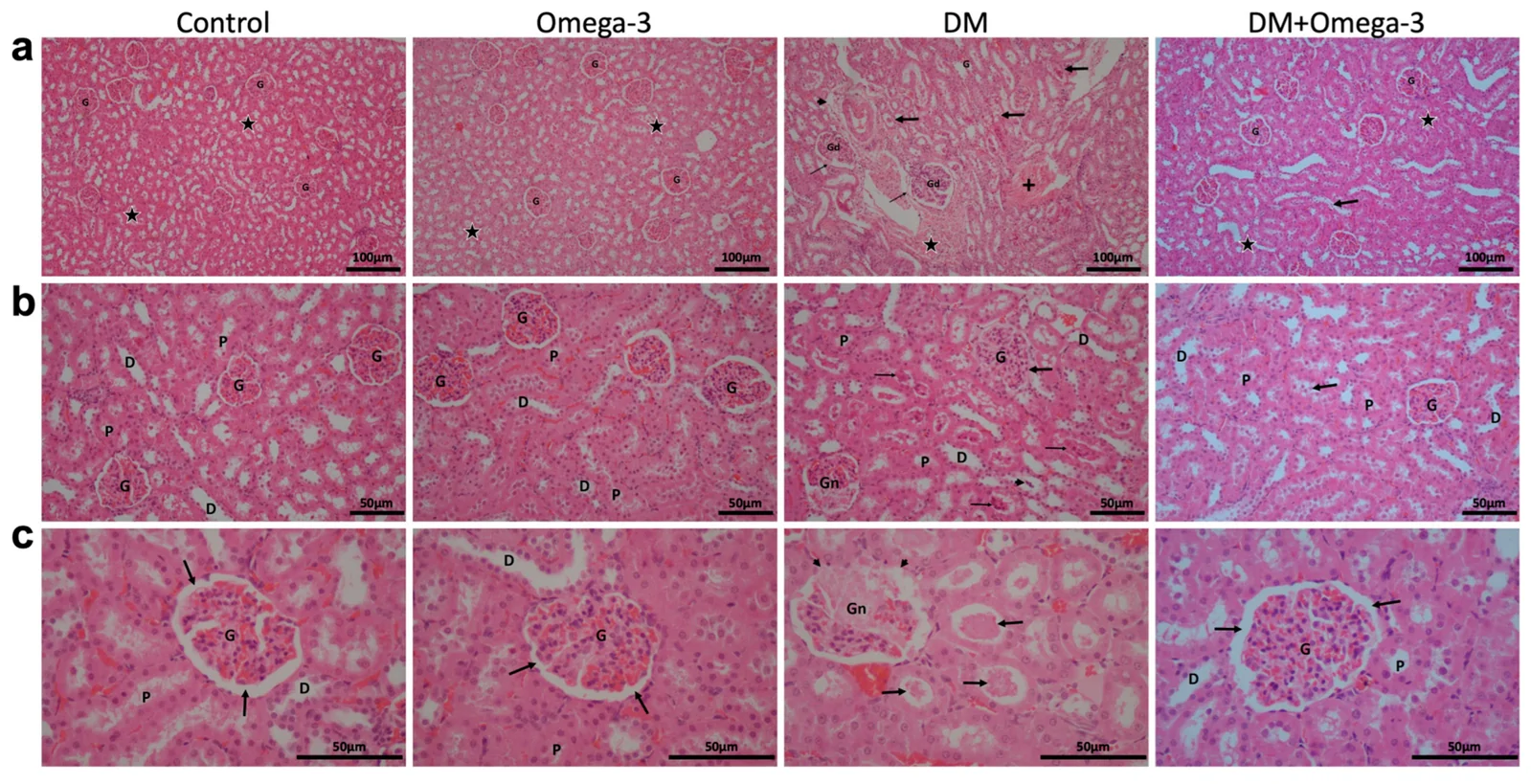
Light microscopic evaluation of renal histopathology across the experimental groups (H&E staining). (**a**) Control group: Normal histological appearance of renal tubules (asterisk) and glomeruli (G). Omega-3 group: Normal histological appearance of renal tubules (asterisk) and glomeruli (G). DM group: Dilatation of the glomeruli (Gd); degeneration of the parietal layer of Bowman’s capsule (thin arrow); tubular degeneration (asterisk); fibrosis (+); tubule damage; degenerated cells in the tubule lumen (thick arrow); and perivascular edema (arrowhead). DM+Omega-3 group: Normal histological appearance of renal tubules (asterisk) and glomeruli (G), with epithelial cell damage in the distal tubule (arrow). (**b**) Control group: Glomeruli (G), distal tubule (D) and proximal tubule (P). Omega-3 group: Glomeruli (G), distal tubule (D) and proximal tubule (P). DM group: Glomeruli (G), proximal tubule (P), distal tubule (D), partial necrosis of the glomeruli (Gn), closure of Bowman’s space (thick arrow) and degenerated cells in the lumen of the proximal and distal tubules (thin arrows). DM+Omega-3 group: Normal histological appearance of renal tubules (asterisk) and glomeruli (G); epithelial cell damage in the proximal tubule (arrow). (**c**) Control group: Glomeruli (G), Bowman’s space (arrow), distal tubule (D) and proximal tubule (P). Omega-3 group: Glomeruli (G), Bowman’s space (arrow), distal tubule (D) and proximal tubule (P). DM group: Partial necrosis of the glomeruli (Gn); degeneration of the parietal layer of Bowman’s capsule (arrowhead); and eosinophilic hyaline deposition in the proximal tubule lumen (arrow). DM+Omega-3 group: Normal histological appearance of the glomeruli (G), Bowman’s space (arrow), the distal tubule (D) and the proximal tubule (P).

Light microscopic examination of H&E-stained kidney sections revealed normal histological architecture of the renal parenchyma in the Control and Omega-3 treated groups, with intact glomeruli and well-organized tubular structures (Figure 3). In contrast, the induction of DM triggered severe histopathological alterations (Figure 3). The glomerular compartments in the DM group exhibited marked dilatation and closure of Bowman’s space, accompanied by degeneration of the parietal layer of Bowman’s capsule (Figure 3). Furthermore, the tubular epithelium in the diabetic kidney demonstrated severe structural degradation, characterized by tubular degeneration, the accumulation of desquamated/degenerated cells in the lumen, and the presence of eosinophilic hyaline casts in the proximal tubules (Figure 3). These parenchymal lesions were consistently accompanied by interstitial expansion and perivascular edema. Remarkably, Omega-3 supplementation (DM+Omega-3 group) significantly mitigated these diabetes-induced structural deformities, preserving both glomerular and tubular integrity. Only mild localized epithelial cell damage persisted in some proximal and distal tubules (Figure 3).

**Figure 4:**
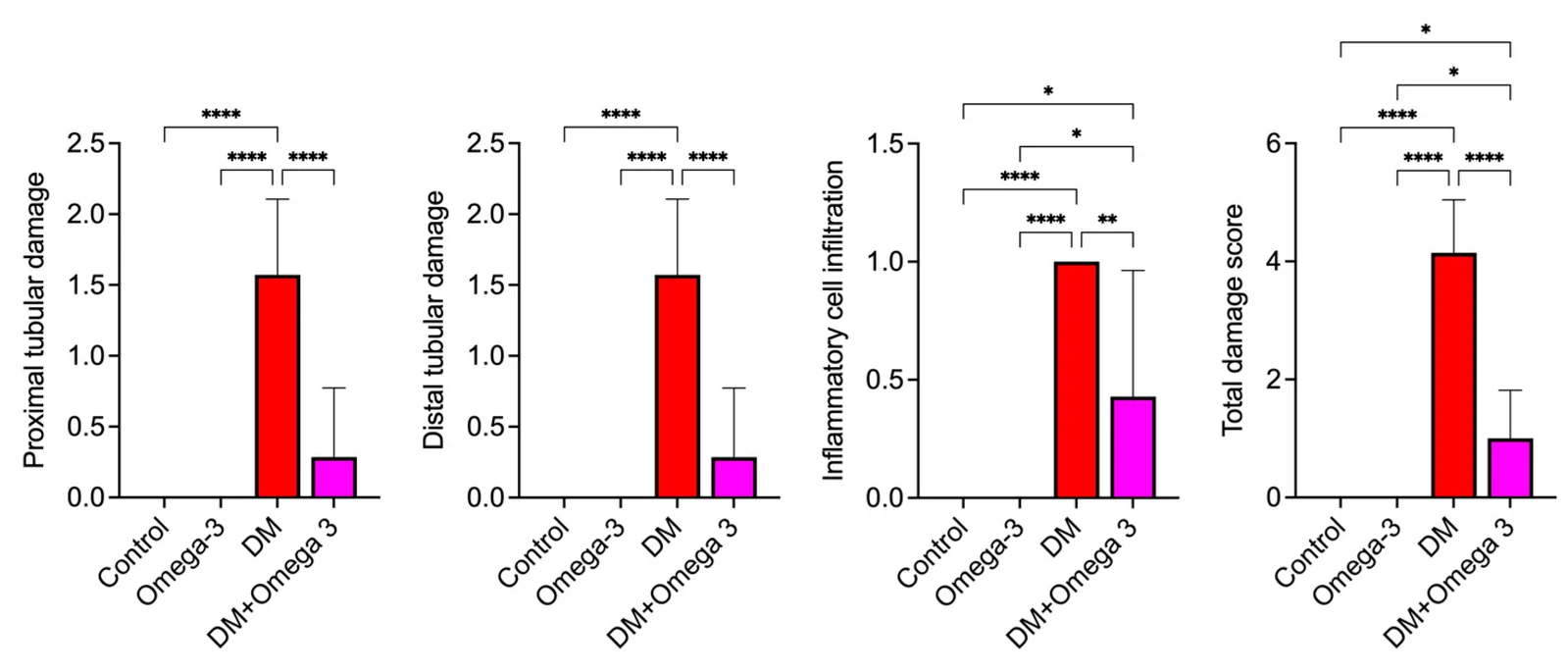
Histological damage scores compared among the groups. Error bars represent the SD. Statistical analysis was performed using one-way ANOVA followed by Tukey’s post-test, with *p* values comparing each pair of experimental groups shown in the graphs. * *p* < 0.05, ** *p* < 0.01, **** *p* < 0.0001.

Semi-quantitative analysis of renal histology showed significant differences between the experimental groups (Figure 4). The DM group showed significant histopathological damage with significantly higher scores for proximal tubular damage (1.62 ± 0.52), distal tubular damage (1.5 ± 0.53) and inflammatory cell infiltration (1 ± 0) compared to the Control and Omega-3 groups, which displayed completely normal histological architecture (0 ± 0) (Figure 4). Thus, the total damage score was high in untreated diabetic rats (4.38 ± 0.74), indicating the extent of renal degeneration due to hyperglycemia. In contrast, in DM+Omega-3, rats showed significantly improved pathological changes. Damage scores for the proximal and distal tubules were decreased to 0.25 ± 0.46 and 0.38 ± 0.52, respectively, with significant suppression of inflammatory infiltration (0.38 ± 0.52). These results suggest that Omega-3 supplementation is effective in reducing the total damage score to 1 ± 0.76, providing significant structural protection against DN (Figure 4), and reducing overall renal injury.

**Figure 5:**
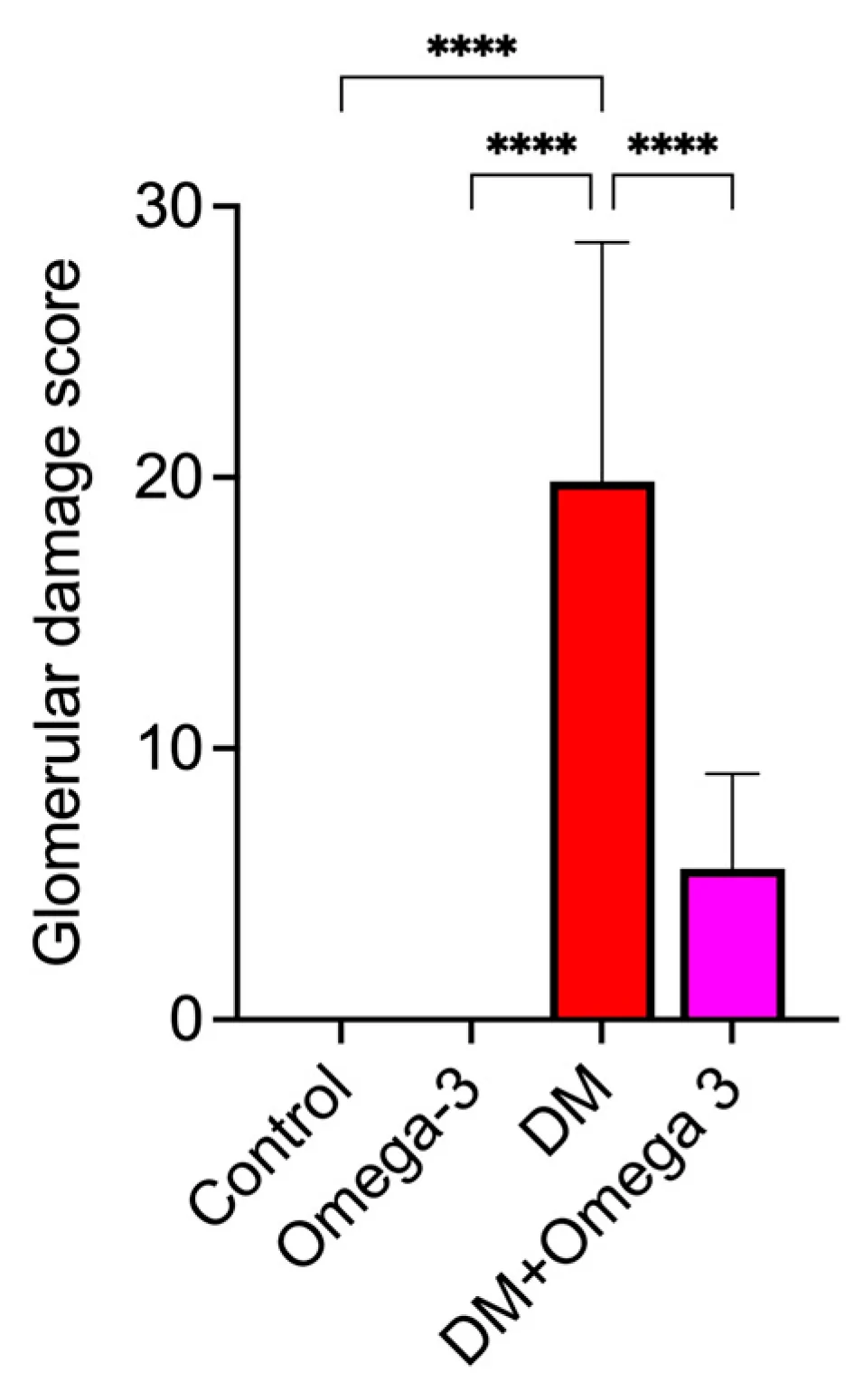
Comparison of total glomerular damage scores across groups. Error bars represent the SD. Statistical analysis was performed using one-way ANOVA followed by Tukey’s post-test, with *p* values comparing each pair of experimental groups shown in the graph. **** *p* < 0.0001.

Similarly, no histopathological changes were observed in the glomerular damage scores in the Control and Omega-3 groups (0 ± 0), whereas a significant increase in glomerular injury was observed in the DM group (19 ± 9.18; *p* = 0.001). Omega-3 supplementation significantly decreased the glomerular damage score in diabetic rats (5.75 ± 3.28), suggesting a notable renoprotective effect via preservation of renal microarchitecture and attenuation of pathological progression (Figure 5).

### 3.4. Immunohistochemical Evaluation of Caspase-3 Expression in Renal Tissue

**Figure 6:**
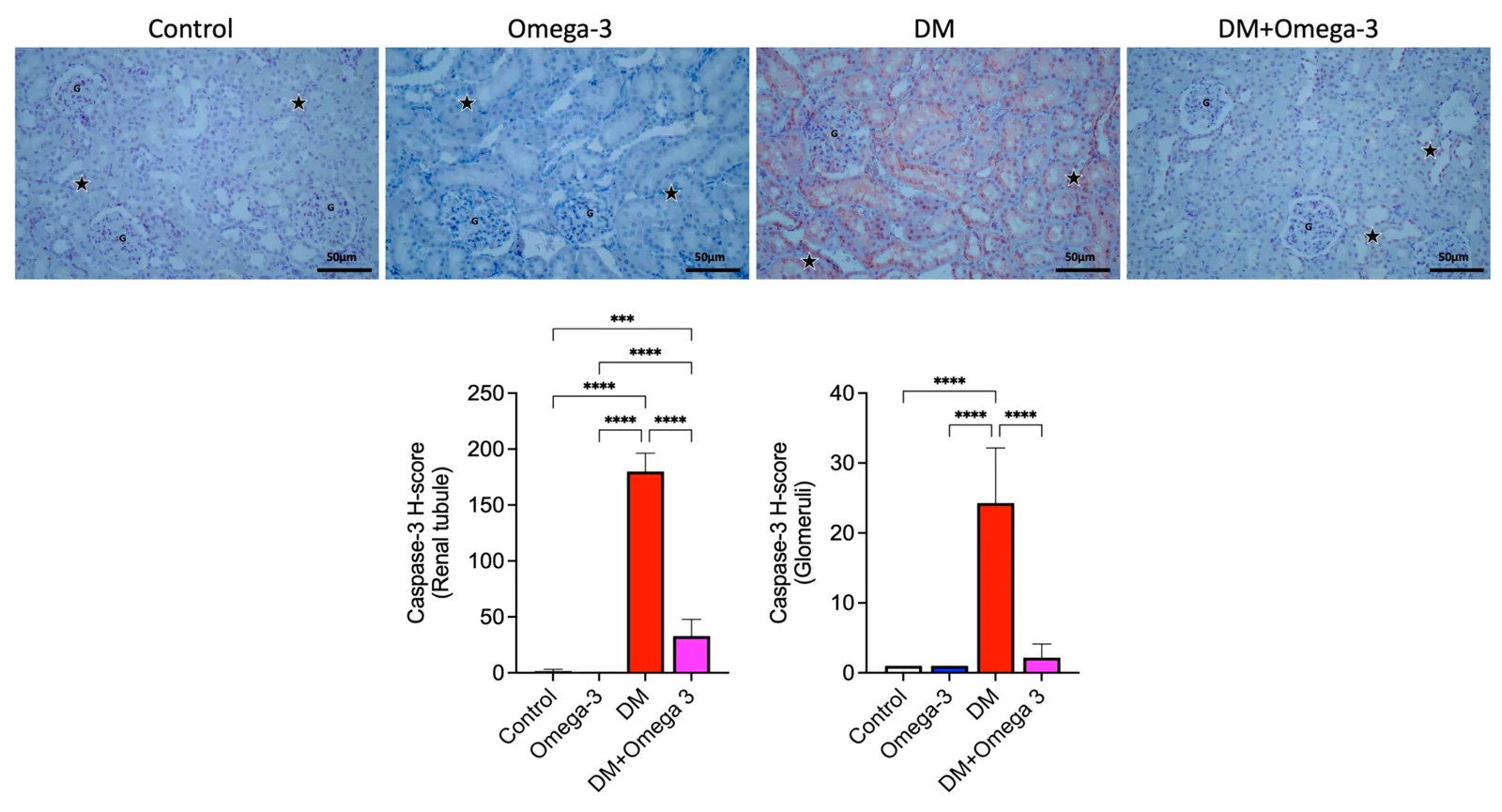
Immunohistochemical analysis of Caspase-3 expression. Control group: Glomeruli (G) and renal tubules (asterisks) show no immunoreactivity. Omega-3 group: Glomeruli (G) and renal tubules (asterisks) without immunoreactivity. DM group: Positive Caspase 3 immunoreactivity in renal tubule epithelial cells (asterisk) and in the glomeruli (G). DM+Omega-3 group: Weak positive Caspase 3 immunoreactivity in renal tubule epithelial cells (asterisk), but no Caspase 3 immunoreactivity in the glomeruli (G). Error bars in graphs represent the SD. Statistical analysis was performed using one-way ANOVA followed by Tukey’s post-test, with *p* values comparing each pair of experimental groups shown in the graphs. *** *p* < 0.001, **** *p* < 0.0001.

Programmed cell death was estimated by measuring Caspase-3 immunoreactivity. Neither the Control group nor the Omega-3 groups showed detectable Caspase-3 expression in either the glomerular or tubular compartments (Figure 6). In contrast, the DM group showed a strong positive Caspase-3 immunoreactivity, primarily in the epithelial cells of the renal tubules and glomeruli, suggesting significant apoptotic activity induced by the diabetic milieu. Omega-3 treatment potently downregulated this pro-apoptotic signaling. The DM+Omega-3 group exhibited a near-complete absence of Caspase-3 immunoreactivity in the glomeruli, with only weak positivity in the renal tubular epithelial cells, indicating potent anti-apoptotic efficacy (Figure 6).

The semi-quantitative analysis of Caspase-3 immunoreactivity, expressed as an H-score, showed significant variations between the experimental groups within both the tubular and glomerular compartments. In the renal tubular compartment, Caspase-3 expression in the renal tubules of the DM group reached its peak intensity (180 ± 16.33), exhibiting a significant increase in pro-apoptotic activity compared to the Control (1.5 ± 1.41) and Omega-3 only (1 ± 0) groups (Figure 6). However, the administration of Omega-3 to diabetic rats (DM+Omega-3) significantly reduced this expression (32.5 ± 13.89), showing a significant decrease in the apoptotic burden of the tubular epithelium (Figure 6). For the glomerular compartment, similar to the tubular findings, the DM group had significantly higher H-scores for Caspase-3 in the glomeruli (22.8 ± 67.56) than the Control and Omega-3 groups, which had insignificant basal expression (10 for both). Interestingly, the DM+Omega-3 group showed almost complete normalization of apoptotic signaling in the glomeruli (2 ± 1.85), which was not significantly different to that in the Control group (Figure 6). These results strongly suggest that the anti-inflammatory and antioxidant properties of Omega-3 contribute to its anti-apoptotic efficacy, thus maintaining the viability of renal cells in the presence of diabetic complications.

### 3.5. Ultrastructural Analysis of Renal Tissue by TEM

**Figure 7:**
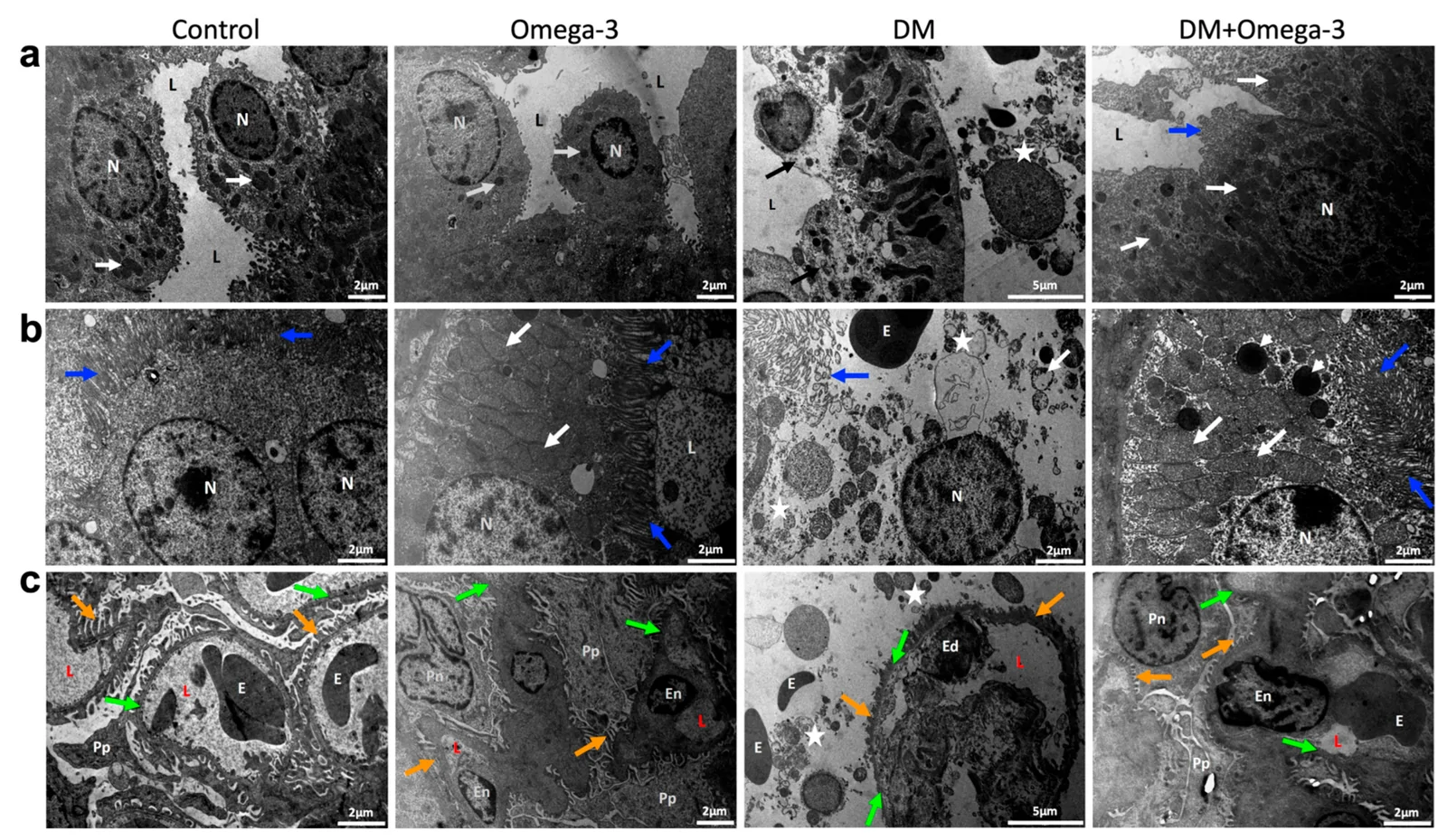
Ultrastructural evaluation of the renal parenchyma via transmission electron microscopy (TEM). (**a**) Control group: Distal tubular epithelial cells exhibit a normal ultrastructural appearance. Features include the nucleus (N), the mitochondria (white arrow) and the distal tubular lumen (black L). Omega-3 group: Distal tubular epithelial cells with a normal ultrastructural appearance. Features include the nucleus (N), mitochondrion (white arrow) and distal tubular lumen (black L). DM group: Distal tubular epithelial cell injury characterized by intracellular edema and organelle degeneration/loss (black arrow), distal tubular lumen (black L) and degenerated cellular debris in the interstitial area (asterisk). DM+Omega-3 group: Distal tubular epithelial cells. Features include the nucleus (N), mitochondrion (white arrow), distal tubular lumen (black L) and dilatation of microvilli (blue arrow). (**b**) Control group: Proximal tubular epithelial cells with a normal ultrastructural appearance. Features include the nucleus (N) and microvilli (blue arrow). Omega-3 group: Proximal tubular epithelial cell with a normal ultrastructural appearance. Features include the nucleus (N), microvilli (blue arrow), mitochondria (white arrow) and proximal tubular lumen (white L). DM group: Necrotic proximal tubular epithelial cell with disrupted integrity and scattered organelles. Features include damaged and degenerated mitochondria (white arrow), degenerated cellular debris (asterisk), a nucleus (N) and dilated, damaged and degenerated microvilli lacking central filament density (blue arrow). DM+Omega-3 group: Proximal tubular epithelial cell. Features include the nucleus (N), microvilli (blue arrow), mitochondria (white arrow) and normal lysosomes (arrowheads). (**c**) Control group: Glomeruli with a normal ultrastructural appearance. Features include capillary lumen (red L), erythrocyte (E), glomerular basement membrane (green arrow), podocyte processes (Pp) and pedicel (orange arrow). Omega-3 group: Glomeruli with a normal ultrastructural appearance. Features include capillary lumen (red L), capillary endothelial nucleus (En), glomerular basement membrane (green arrow), podocyte processes (Pp), podocyte nucleus (Pn) and pedicel (orange arrow). DM group: Features include degenerated glomerular capillary endothelium (Ed), capillary lumen (red L), damaged glomerular basement membrane (green arrow), degenerated pedicel remnants (orange arrow), degenerated cellular debris (asterisk) and erythrocyte (E). DM+Omega-3 group: Glomeruli with a normal ultrastructural appearance. Features include capillary lumen (red L), capillary endothelial nucleus (En), glomerular basement membrane (green arrow), podocyte processes (Pp), podocyte nucleus (Pn), pedicel (orange arrow) and erythrocyte (E).

The ultrastructural evaluation by TEM provided insight into the nephroprotective mechanisms of Omega-3. The ultrastructure of the distal and proximal tubules in the Control and Omega-3 groups were normal, with normal nuclei, intact mitochondria and well-preserved apical microvilli. The GFB was intact in these groups, and normal capillary endothelia, a uniform GBM and regular podocyte pedicels (foot processes) were observed (Figure 7). The DM group showed severe ultrastructural deterioration. Proximal tubule cells showed apparent necrosis, characterized by the destruction of cellular integrity, dispersed cytoplasmic organelles and severely damaged mitochondria. Notably, the apical microvilli of the proximal tubules were degenerated, dilated and lacked central filament density. The distal tubules developed intracellular edema and organelle loss. Cellular debris accumulated in the interstitial space. Severe damage to the GBM, capillary endothelium and dramatic degeneration and effacement of podocyte pedicels was observed in the glomeruli of diabetic rats (Figure 7). In contrast, Omega-3 intervention in the DM+Omega-3 group significantly restored these ultrastructural parameters (Figure 7). Proximal and distal tubular cells recovered morphological stability, with normal nuclei and healthy mitochondria, although some microvilli showed minor residual dilatation. Most importantly, Omega-3 preserved the ultrastructural integrity of the glomerular filtration barrier, with normal capillary endothelium, intact GBM and healthy podocyte pedicels (Figure 7).

## 4. Discussion

The present study provides a detailed histological, ultrastructural and biochemical analysis of the protective efficacy of Omega-3 fatty acids against DN in a STZ-induced rat model. Our main findings show that Omega-3 supplementation significantly reduces hyperglycemia-induced renal parenchymal injury and inhibits Caspase-3-mediated apoptotic signaling, thus maintaining the structural integrity of the GFB and tubular compartments.

DN is characterized by a progressive decline in GFR and the accumulation of nitrogenous waste products [16]. Our results demonstrated elevated BUN and creatinine levels in the DM group, suggesting potential renal impairment. However, as direct GFR was not calculated, caution must be exercised when defining absolute uremia. Furthermore, the structural integrity of the GFB was preserved by Omega-3 supplementation, as evidenced by TEM analysis showing reduced podocyte effacement and an intact basement membrane. This was aligned with significantly lower levels of biochemical markers in the DM+Omega-3 group.

Renoprotection is closely related to the modulation of oxidative stress [17]. Chronic hyperglycemia leads to the overproduction of ROS via the polyol pathway and non-enzymatic glycation processes [18]. Our biochemical data, which show increased MDA and TOS in diabetic rats alongside decreased SOD, CAT and GSH, are consistent with Brownlee’s “unifying hypothesis” of diabetic complications [19]. Omega-3 fatty acids, including EPA and DHA, act as potent ligands for the G protein-coupled receptor 120 (GPR120) and peroxisome proliferator-activated receptors (PPARs) that orchestrate the antioxidant defenses. Our results support the idea that Omega-3 supplementation restores redox homeostasis and prevents lipid peroxidation and tissue damage [20].

Results of renal tissue SOD activities that are statistically similar to those of the control group are often described in the literature as a transient adaptive compensation mechanism against chronic hyperglycemia in models of STZ-induced diabetic nephropathy. This paradox can be explained by the fact that, in the early and intermediate stages of diabetes, an increase in ROS activates the Nrf2 signaling pathway, leading to the increased expression of antioxidant genes. However, the simultaneous dramatic increase in MDA and TOS values, even with these “pseudo-normal” enzyme levels, suggests that the existing SOD activity is insufficient to prevent massive lipid peroxidation and ultrastructural damage within the tissue [21,22]. Based on the current literature, the enzymatic stability indicates a “transition phase” in which the catalytic function of the enzyme is compromised by non-enzymatic glycation. However, supplementation with exogenous antioxidants, such as Omega-3, restores this weak defense system and maintains structural integrity [23].

In our study, light microscopic and ultrastructural analysis clearly demonstrated the significant renoprotective effects of Omega-3 supplementation. The DM group exhibited typical features of advanced DN, such as glomerular dilatation, obliteration of Bowman’s space, and intraluminal accumulation of hyaline casts. These features are indicative of plasma protein leakage resulting from tubular dysfunction.

Glomerular damage scores showed significant glomerular injury in the DM group, contrasting with the intact architecture observed in the non-diabetic groups. This condition involves the activation of the TGF-β1/Smad signaling pathway in response to chronic hyperglycemia, which leads to mesangial expansion and glomerulosclerosis [24]. Supplementation with Omega-3 significantly reduced glomerular damage scores by around 69.7% in diabetic rats. This finding is consistent with experimental studies showing that Omega-3 monotherapy can significantly decrease oxidative stress, inflammation and key fibrogenic mediators, such as TGF-β1 and Caspase-3 in STZ-induced diabetic nephropathy [25]. This finding is also supported by clinical data indicating that Omega-3 supplementation can slow the progression of the disease in patients with early-stage diabetic nephropathy [26]. However, the presence of a statistically significant difference between the DM+Omega-3 group and the non-diabetic control group suggests that 42 days of supplementation was insufficient for full glomerular recovery, and that longer treatment periods may be required for complete restoration of glomerular microarchitecture in well-established diabetic nephropathy.

In the context of the podocyte–GBM complex, our TEM findings were particularly insightful. Ultrastructural correlates of proteinuria included the severe effacement of podocyte pedicels (foot processes) and the thickening of the GBM, which was observed in the DM group. As podocytes are terminally differentiated cells, their loss or detachment represents a critical “point of no return” in DN [27]. Remarkably, the DM+Omega-3 group demonstrated almost complete restoration of podocyte architecture. This suggests that Omega-3 may stabilize the podocyte cytoskeleton, by modulating nephrin expression or inhibiting the TGF-β1/Smad signaling pathway, which is the main cause of podocyte damage and interstitial fibrosis in diabetic conditions [28].

Another important finding of our study was the significant decrease in Caspase-3 immunoreactivity in the DM+Omega-3 group. Tubular epithelial cells and podocytes undergo apoptosis, which is a major contributor to renal atrophy in patients with DM [29]. The present study’s key finding was the marked decrease in Caspase-3 immunoreactivity in the DM+Omega-3 group, suggesting the powerful anti-apoptotic effect of Omega-3 PUFAs against diabetic insults. Programmed cell death is a hallmark of DN, defined as the progressive loss of tubular epithelial cells and podocytes. It is the main driver of renal atrophy and the subsequent decrease in the glomerular filtration rate [16,30]. Our data suggest that these harmful effects are primarily orchestrated via the activation of the intrinsic (mitochondrial) apoptosis pathway by the diabetic milieu. Our ultrastructural analysis further confirmed this link; TEM revealed severe mitochondrial pathology, including swelling and loss of cristae integrity. These structural disruptions lead to the permeabilization of the outer mitochondrial membrane and translocation of cytochrome c into the cytosol. At the time of release, cytochrome c induces the formation of the apoptosome complex. This complex then which proteolytically activates executioner Caspase-3, thereby completing the apoptotic cascade [31,32].

The dramatic downregulation of Caspase-3 expression in Omega-3-treated rats shows the ability of these fatty acids to maintain mitochondrial membrane stability and to reduce the pro-apoptotic signaling induced by chronic hyperglycemia [25]. Indeed, Omega-3 effectively intercepts the transition from metabolic stress to irreversible cellular loss by protecting the mitochondrial architecture, as demonstrated by the restored cristae morphology observed in the treatment group. Furthermore, given the improvement in oxidative stress parameters (e.g., decreased MDA and conserved SOD levels) observed in our study, it is highly probable that Omega-3 stabilizes this apoptotic cascade mainly by limiting the overproduction of mitochondrial ROS. This synergistic coupling of antioxidant and antiapoptotic mechanisms provides a robust cytoprotective strategy for managing diabetic renal complications. The diabetic environment triggers the intrinsic (mitochondrial) apoptotic pathway with mitochondrial dysfunction. This is demonstrated by our TEM results showing swollen and disrupted cristae, which lead to the release of cytochrome c and the activation of executioner Caspase-3 [33].

The significant decrease in H-score from high (180 in tubules) in the DM group to low (32.5) in the DM+Omega-3 group, shows the potent anti-apoptotic effects of polyunsaturated fatty acids (PUFAs). This is likely due to the incorporation of Omega-3 into mitochondrial membranes, which increases mitochondrial membrane stability and reduces the pro-apoptotic Bax/Bcl-2 ratio. This mechanism is frequently mentioned in the literature concerning Omega-3 and metabolic syndrome [34].

Interestingly, blood glucose decreased over time in the DM+Omega-3 group, falling from 352 mg/dL to 180 mg/dL by day 42. Although the intra-group variance was high, the numerical trend suggests that Omega-3 may improve systemic insulin sensitivity or protect pancreatic β-cells against glucotoxicity. This metabolic stabilization likely synergizes with the localized antioxidant effects in the kidney. Partial glycemic control is known to significantly retard the progression of diabetic microvascular complications, which supports the clinical relevance of our findings.

## 5. Conclusions

In conclusion, the present study provides evidence that Omega-3 fatty acids have a significant renoprotective effect in diabetic rats. This is achieved by preserving the GFB and preventing podocyte pedicel effacement. It also maintains tubular epithelial integrity, reduces proteinaceous cast formation and suppresses the Caspase-3-mediated apoptotic cascade within the renal parenchyma. Furthermore, it reverses the oxidative stress burden by reactivating endogenous antioxidant enzymes.

While these findings suggest that Omega-3 could be an effective adjuvant therapeutic agent, further preclinical investigations utilizing extended treatment periods and varying doses are critically required to establish the precise duration and dose needed for complete structural restoration before clinical translations can be considered.

## 6. Strengths, Limitations and Future Directions

A major strength of this study is its multimodal design, which combines biochemical, semi-quantitative histopathological, immunohistochemical and TEM analysis. This enabled a comprehensive evaluation of renal injury at the systemic to ultrastructural level. Additionally, the blinded histopathological assessment increased the reliability of the methodological findings. However, some limitations of this study should be acknowledged. The 42-day experimental duration may not have been sufficient to reproduce the chronic fibrotic and sclerotic changes that are characteristic of advanced human diabetic nephropathy. Additionally, the present study did not evaluate early biomarkers of renal injury, such as Cystatin C, $\beta_{2}$-microglobulin and kidney injury molecule-1 (KIM-1), or the glomerular filtration rate, due to technical constraints. Thus, future long-term studies incorporating these sensitive markers are required to better elucidate the hyper-acute phases of diabetic nephropathy. While the present findings support the antioxidant and anti-apoptotic effects of Omega-3 fatty acids, the molecular mechanisms involved remain unclear. Further studies on the Nrf2/HO-1 pathway and the specialized pro-resolving lipid mediator pathway, as well as longitudinal clinical trials, are needed to clarify the therapeutic potential of Omega-3 supplementation in diabetic nephropathy.

## Data Availability

All data are available within this manuscript. For further inquiries, please contact the corresponding author.

## Abbreviations

The following abbreviations are used in this manuscript:

BUN	Blood Urea Nitrogen
DM	Diabetes Mellitus
DN	Diabetic Nephropathy
GFB	Glomerular filtration barrier
GSH	Glutathione
MDA	Malondialdehyde
OSI	Oxidative Stress Index
STZ	Superoxide Dismutase
SOD	Streptozotocin
TAS	Total Antioxidant Status
TEM	Transmission Electron Microscopy
TOS	Total Oxidant Status
